# Circadian Typology and Physical Activity in Young Adults with Gaming Disorder

**DOI:** 10.3390/medicina60121950

**Published:** 2024-11-26

**Authors:** Tien-Wei Hsu, Ju-Yu Yen, Wei-Chiang Yeh, Chih-Hung Ko

**Affiliations:** 1 Graduate Institute of Clinical Medicine, College of Medicine, Kaohsiung Medical University, Kaohsiung 807, Taiwan; 2 Department of Psychiatry, E-DA Dachang Hospital, I-Shou University, Kaohsiung 807, Taiwan; ed109912@edah.org.tw; 3 Department of Psychiatry, E-DA Hospital, I-Shou University, Kaohsiung 824, Taiwan; 4 Department of Psychiatry, Kaohsiung Municipal Ta-Tung Hospital, Kaohsiung Medical University, Kaohsiung 801, Taiwan; 5 Department of Psychiatry, Faculty of Medicine, College of Medicine, Kaohsiung Medical University, Kaohsiung 807, Taiwan; 6 Department of Psychiatry, Kaohsiung Medical University Hospital, Kaohsiung Medical University, Kaohsiung 807, Taiwan; 7 Department of Psychiatry, Kaohsiung Municipal Siaogang Hospital, Kaohsiung Medical University, Kaohsiung 812, Taiwan

**Keywords:** gaming disorder, delayed sleep phase syndrome, physical activity, sleep pattern, actigraphy

## Abstract

*Background and Objectives*: Exploring daily lifestyle characteristics in individuals with gaming disorder (GD) might identify underlying causes and intervention points. However, integrative and subjective assessments are lacking in studies on this topic. This study aimed to assess circadian typology and physical activity in young adults with GD. *Materials and Methods*: We recruited 60 participants with GD and 120 age- and sex-matched controls. GD and delayed sleep phase syndrome (DSPS) were diagnosed through structured interviews. Physical activity and sleep patterns were measured using actigraphy. Self-reported measures were chronotype and insomnia by using composite scale of morningness (CSM) and the Pittsburg insomnia rating scale (PIRS). *Results*: We found that DSPS and eveningness chronotype was more prevalent in the GD group than in the control group. The GD group also contained more participants with insomnia with higher PIRS and a longer time to fall asleep compared with the control group. The GD group had lower physical activity levels for daily calorie expenditure, daily steps, and daily walking distance compared with the control group. No significant differences were observed in body weight and sleep duration between these groups. *Conclusions*: Individuals with GD, compared to HC, exhibit an eveningness chronotype, poorer sleep quality, lower physical activity, and higher obesity risk, suggesting that lifestyle adjustments like increased exercise and earlier sleep might help modify habitual behaviors and potentially further provide a way to treat GD.

## 1. Introduction

Problematic gaming has been extensively studied in the fields of psychology and psychiatry for the past two decades [1,2,3]. Diagnostic and Statistical Manual of Mental Disorders, Fifth Edition provides specific criteria that are used to diagnose internet gaming disorder (IGD) [4]. The International Classification of Diseases, 11th Revision (ICD-11) defines gaming disorder (GD) [5] as addictive behavior and identifies hazardous gaming as a health-related problem. GD is characterized by excessive and compulsive engagement in online games, leading to disruptions in daily life, responsibilities, and relationships [4]. Psychological factors associated with GD include depression, anxiety, loneliness, impulsivity, and escape motivation [6,7]. Exploring the daily lifestyle characteristics of individuals with GD may offer insight into underlying causes or potential interventions.

Internet addiction is associated with delayed circadian rhythms, insomnia, low sleep quality, and short sleep duration [8,9,10]. Circadian rhythm disorder refers to a group of conditions where the body’s internal clock, which regulates the 24 h cycle of biological processes (such as sleep–wake patterns), is misaligned with the external environment or a person’s desired schedule [11]. Delayed sleep–wake phase (DSPS) is characterized by a consistent delay in sleep onset and wake times, often causing difficulty waking up in the morning [11]. Insomnia disorder is a common sleep disorder characterized by difficulty initiating or maintaining sleep, or experiencing non-restorative sleep, despite having adequate opportunity to sleep [12]. Being overweight and having a low level of physical activity is also associated with internet addiction [13,14,15,16]. Evidently, several aspects of a person’s daily lifestyle are related to internet addiction. However, few studies have examined these characteristics together. Furthermore, most of the aforementioned studies have certain limitations. First, they assessed internet addiction or GD by using subjective or self-reported inventories and lacked expert-conducted structured interviews for confirming diagnoses. Second, they assessed sleep patterns and physical activity levels subjectively and did not use objective measurements. Lastly, they have defined obesity using body mass index (BMI) values and without using body fat percentage and consequences of obesity, such as blood pressure, or blood lipid concentration.

This clinical study assessed sleep patterns, body weight and related measurements, and physical activity using objective and subjective assessments. We further focused on GD rather than internet addiction to ensure the homogeneity of participants and existing diagnostic criteria [5]. In addition, this study focused on the young adult population because many previous studies on GD have centered on children and adolescents, with relatively few addressing young adults [17,18]. We aim to investigate the lifestyle of individuals who continue to exhibit GD patterns even after entering university or starting work, at a stage when their brain development is more mature. Beyond understanding the relationship between these measurements and GD, this information may provide insights and entry points for future treatment and intervention strategies for GD.

## 2. Materials and Methods

### 2.1. Participants

We recruited participants between May 2019 and October 2020. We advertised the study by posting an advertisement on the bulletin board system of a university. Adults aged between 20 and 40 who had completed more than 12 years of education were eligible for inclusion. Participants underwent a semi-structured interview with a senior experienced board-certified psychiatrist. GD was defined using ICD-11 criteria. The Chinese version of the Mini International Neuropsychiatric Interview [19] was used to identify psychiatric comorbidities. Finally, we included 60 individuals with GD and 120 healthy controls (HC). The GD group had an average age of 26.42 years (standard deviation, SD = 4.54) with a male ratio of 76.7% (46/60), while the HC group had an average age of 27.18 years (SD = 4.56) and the same male ratio of 76.7% (92/120). The GD group had an average education level of 15.80 years (SD = 1.36), compared to 16.02 years (SD = 1.68) in the HC group (Table 1). Participants were excluded if they had a psychotic disorder, bipolar I disorder, substance use disorder, intellectual disability, or brain injury. Medical and school histories were reviewed. Each participant with GD was matched with two sex- and age-matched (±3 years) controls. The Institutional Review Board of Kaohsiung Medical University Hospital approved the study protocol (KMUHIRB-SV-II-20170073). All methods were performed in accordance with the Declaration of Helsinki.

### 2.2. Measurements

#### ICD-11 GD Criteria

The diagnostic criteria for GD were as follows: (1) impaired control over gaming habits, (2) increased priority given to gaming, and (3) gaming for >25 h per week [20]. Individuals for whom these behavioral patterns caused considerable functional impairment over >12 months were considered to have GD. Further details are provided in another study [21]. Criteria were considered relevant only when they were persistent and clinically significant in accordance with the recommendations of the ICD-11 [5].

Participant demographics: We collected demographic data on age, sex, years of education, height, weight, BMI, body fat percentage, and body weight. We used a body composition analyzer (HBF-370, Omron Co. LTD., Kyoto, Japan). Participants with BMI ≥ 30 were considered obese. Participants with BMI between 25 and 30 were considered overweight [22].

Chinese Internet Addiction Scale (CIAS): A widely used self-report questionnaire designed to assess the severity of internet addiction, particularly among adolescents and young adults. It measures five dimensions of internet addiction, including compulsive use, withdrawal, tolerance, interpersonal and health problems, and time management issues [8]. The total scores range from 0 to 100, with higher scores indicating more severe internet addiction. A score of 63 points would be used as the diagnostic cut-off point [2].

Delayed sleep phase syndrome (DSPS): A psychiatrist interviewed all participants to ascertain whether (1) their preference in eveningness (eveningness chronotype) was fixed; (2) they had difficulty falling asleep if they had tried to sleep earlier; and (3) they had trouble sleeping when their sleep pattern was disrupted. Participants who answered positively to these questions and who went to bed later than 1 a.m. and woke up later than 9 a.m. were categorized as having DSPS. The diagnostic criteria of DSPS were modified from those of delayed sleep–wake phase disorder of the International Classification of Sleep Disorders, Third Revision (ICSD-3) [23].

Composite scale of morningness: The composite scale of morningness (CSM) is a self-assessment used to evaluate an individual’s chronotype, which is a person’s natural inclination with regard to the times of day when they are most active. The CSM contains 13 items [24]. Individuals can be categorized into three groups based on their CSM scores, which range from 13 to 55, with extreme eveningness at the lower end and extreme morningness at the higher end of the scale. CSM scores of ≥44, between 23 and 43, and ≤22 indicate morningness, intermediate, and eveningness chronotypes, respectively [24].

Pittsburg insomnia rating scale—20-item version: The Pittsburgh insomnia rating scale—20-item version (PIRS) assesses the severity of insomnia symptoms [25]. The PIRS contains 20 items ranked on a 4-point Likert-type scale that covers aspects related to difficulty falling asleep, staying asleep, waking up too early, and the effects of insomnia on daytime functioning. The total scores range from 0 to 60, with higher scores representing more severe insomnia. We used a cutoff score of 20 to define clinical insomnia [25]. The test–retest reliability and Cronbach’s alpha for the PIRS-20 were 0.92 and 0.95, respectively [26].

Lipid profile and blood pressure: We measured high-density lipoprotein, low-density lipoprotein, triglycerides, total cholesterol, and blood pressure in the morning after an overnight fast of 8 h.

Actigraphy: Participants wore an accelerometer (JSmax SB-V11, Jing Shin Technologies, Tainan, Taiwan) on their left wrist 24 h a day for 1 week to measure daily steps, daily walking distance, estimated calorie expenditure, time to fall asleep, and sleep duration.

### 2.3. Statistical Analysis

Data were analyzed using SPSS, version 25 (IBM, New York, NY, USA). For between-group comparisons, continuous variables were examined using the independent *t*-test, and nominal variables were assessed using Pearson’s χ^2^ test. Statistical significance was defined by two-tailed *p* ≤ 0.05.

## 3. Results

In total (Table 1), 180 participants were enrolled, among which 60 participants were categorized into a GD group and 120 into a healthy control (HC) group. Relative to the HC group, the GD group had a lower CSM score (GD: 25.22 [6.56]; HC: 34.91 [6.67], *p* < 0.001), a higher CIAS score (GD: 79.48 [11.62]; HC: 34.91 [6.67], *p* < 0.001), and a higher PIRS score (GD: 28.85 [10.88]; HC: 14.88 [9.48], *p* < 0.001; Table 1). Relative to the HC group, the GD group included a larger number (proportion) of individuals with DSPS (GD: 32 [53.33%]; HC: 11 [9.17%], *p* < 0.01) and insomnia (GD: 17 [28.33%]; HC: 8 [7.14%], *p* < 0.01). As indicated by CSM scores, the GD group was more likely to have an eveningness chronotype than the HC group (*p* < 0.001). No significant differences were observed between the groups in terms of BMI, body fat percentage, blood pressure, or lipid profile. Notably, the GD group included a larger number of obese individuals (10 [16.70%]) than the HC group (9 [7.5%]). 

In actigraphic analysis (Table 2), the GD group exhibited lower daily estimated calorie expenditure (GD: 305.94 [150.54]; HC: 469.69 [159.02], *p* < 0.001, kilocalorie), fewer daily walking steps (GD: 5865.74 [2892.53]; HC: 8865.00 [2904.64], *p* < 0.001, number of steps), and shorter daily walking distance (GD: 4.64 [2.30]; HC: 7.15 [2.45], *p* < 0.001, kilometer) than the HC group. The GD group took more than twice as long as the HC group to fall asleep (GD: 134.09 [70.74]; HC: 65.15 [60.11], *p* < 0.001, min); however, no significant difference was observed in sleep duration between the groups (*p* = 0.057).

We next divided the GD group into two subgroups (Table 3): DSPS (*n* = 32) and non-DSPS (*n* = 28; Table 3). The DSPS group had lower CSM scores than the non-DSPS group (DSPS: 22.06 [4.75]; non-DSPS: 28.82 [6.54], *p* < 0.001), indicating that the DSPS group was more likely to have an eveningness chronotype than the non-DSPS group (DSPS: 17 [53.10%]; non-DSPS: 5 [17.90%], *p* = 0.005). In addition, the DSPS group was more likely to have insomnia than the non-DSPS group (DSPS: 14 [43.80%]; non-DSPS: 3 [10.70%], *p* = 0.005). However, regarding mean CIAS and PIRS scores, there were no significant differences between the DSPS group and non-DSPS group (CIAS, DSPS: 80.32 [12.59], non-DSPS: 78.75 [10.85], *p* = 0.605; PIRS, DSPS: 30.09 [10.47], non-DSPS: 27.43 [11.34], *p* = 0.348).

The non-DSPS group had a higher average BMI than the DSPS group (DSPS: 22.33 [4.04]; non-DSPS: 26.47 [5.29], *p* = 0.001) and was more likely than the DSPS group to be overweight or obese (*p* = 0.013, Table 3). No significant differences were observed in other measures, including body fat percentage, blood pressure, and lipid profile between these two groups. None of the recorded actigraphic measurements significantly differed between these two groups (Table 4).

## 4. Discussion

The present study compared the sleep patterns and physical activity of young adults with and without GD. Our main findings are as follows. First, eveningness chronotype and DSPS were more prevalent in the GD group than in the HC group. Second, the GD group had poorer sleep quality than the HC group and took longer to fall asleep, and insomnia was more prevalent in the GD group than in the HC group. However, sleep duration did not significantly differ between the groups. Third, the GD group was less physically active and more likely to be obese than the HC group. Fourth, in the GD group, no significant differences between participants with and without DSPS were observed in psychiatric and actigraphic measures, except for obesity and insomnia, which were less and more prevalent, respectively, in those with DSPS.

The sleep patterns we observed in the GD group, including eveningness chronotype and insomnia, are consistent with the findings of other studies [9,27]. Notably, our findings were determined using objective instruments, including actigraphy and interviews with a psychiatrist, rather than subjective inventories typically used in other studies. Several potential causes exist for sleep problems among individuals with GD. First, screens emit blue light, which blocks melatonin secretion. Individuals playing games on screens that emit blue light are being exposed to blue light. Continuous exposure to blue light, especially at night, may contribute to difficulty in falling asleep and may disrupt the circadian rhythm [28]. Second, in this study, the GD group exhibited higher attention deficit–hyperactivity disorder symptoms than the control group. Early insomnia and eveningness chronotype are characteristics of attention deficit–hyperactivity disorder [29,30], and this finding was also supported by genome studies [29,31]. Lastly, eveningness chronotype is associated with various addictive disorders, including substance abuse disorder [32]. This association may be mediated by genes associated with circadian rhythm. For example, alcohol-dependent male subjects showed reduced mRNA levels of the CLOCK gene compared to control subjects [33]. Single nucleotide polymorphisms in the CLOCK gene are associated with addiction [31]. The T allele of rs2412648 is associated with alcohol dependence [34], and the G allele of rs11240 is associated with alcohol use disorder and depression [35]. The present study observed no differences in sleep duration between the GD and HC groups, whereas a meta-analysis revealed a shorter sleep duration in patients with problem gaming than in those without problem gaming [10]. However, most of the studies included in that meta-analysis used self-reported data on sleep duration, which we do not consider objective. Clinical studies that use accurate and objective measurement tools and larger sample sizes are warranted to investigate sleep duration in individuals with GD.

We can readily assume that individuals with internet addiction spend a large amount of time in front of screens, which can lead to reduced physical activity and an increased risk of obesity. Internet addiction is associated with obesity [14,16,36]. In the present study, no significant differences were observed between the GD and HC groups in BMI or related metabolic measures, including blood pressure, lipid profile, and body fat percentage. However, the GD group was more likely to be obese (BMI > 30) than the HC group. Regarding physical activity, the relationship between internet addiction and reduced physical activity is unclear. Several studies have found no association [37,38], whereas a Turkish study observed an inverse correlation between internet addiction and walking distance [13], which is consistent with the findings of the present study. Notably, the aforementioned studies have assessed physical activity by using self-reported questionnaires [13,37,38]. In contrast, our study assessed physical activity by using actigraphy, which is more objective. In summary, individual differences may exist. Some internet addicts may engage in high levels of physical activity, and some non-addicts may lack engagement in physical activity. Other factors may influence physical activity levels and body weight. For example, coping style was found to mediate the relationship between internet addiction and obesity [39].

Several studies have observed a relationship between eveningness chronotype and internet addiction [9,27]. We assessed insomnia severity in participants with GD, and the results revealed no significant difference between those with and without DSPS, both subjectively and objectively. A Norwegian study of 50 054 students found that DSPS was associated with obesity, reduced physical activity, sleep problems, and increased time to fall asleep [40]. In the present study, among participants with GD, DSPS was associated with insomnia, but not with physical activity. Furthermore, among participants with GD, those with DSPS were less likely to be obese or overweight than those without DSPS. However, it is important to note that the present study assessed participants within the GD group, and these participants had relatively severe insomnia and low CSM scores, whereas the Norwegian study assessed DSPS in the general population [40]. Therefore, our comparison is more likely to not show significant differences. Additionally, our subgroup analysis is limited by the small sample size in this group, which reduced the statistical power of this study.

Our study has several limitations. First, the relatively small sample size limited the statistical power of our analyses, especially in the subgroup analysis. The sample size was limited by the fact that not every participant underwent actigraphy. Second, calorie expenditure may have been underestimated because the actigraphy unit was worn on the wrist, and the movement of the wrist may not correspond to whole-body movement. For example, the actigraphy unit is not capable of registering movement during certain activities, such as push-ups. Third, we did not assess dietary habits; therefore, we could not conduct a comprehensive assessment of lifestyle characteristics. Fourth, other sleep parameters (such as wake after sleep onset or sleep efficacy) and physical activity parameters (such as interdaily stability, intradaily variability, or relative amplitude) were not measured and collected, which limited our analyses. Fifth, we used nonstandard criteria to diagnose DSPS. We used the modified criteria for delayed sleep–wake phase disorder outlined in the ICSD-3 to define DSPS. Strictly adhering to the ICSD-3 criteria would have necessitated that we arrange polysomnography to rule out other sleep disorders, which would have been challenging for our study settings. Sixth, potential biases related to subjective self-assessment questionnaires could not be avoided. Lastly, we did not gather detailed socioeconomic information about our participants, such as income, residence, family history of psychiatric diseases, or types of employment, which might potentially mitigate our results. Therefore, our results may not be generalizable to other research or clinical contexts. Finally, this is a cross-sectional study; therefore, causal relationships cannot be inferred.

## 5. Conclusions

In conclusion, the present study assessed sleep, physical activity, and weight management patterns in individuals with GD and matched controls by using subjective and objective measurement tools. Compared with HC, individuals with GD tended to have an eveningness chronotype, poorer sleep quality, lower physical activity levels, and a higher risk of obesity. Lifestyle adjustments, such as exercise and sleeping earlier, may serve as a way to modify habitual behaviors in individuals with GD and have the potential to further alleviate GD symptoms.

## Figures and Tables

**Table 1:** Demographic, clinical, and psychiatric characteristics of participants with and without gaming disorder.

	GD Group (*n* = 60)	Control Group (*n* = 120)	*p* Value
Demographic characteristics			
Male (%)	46 (76.7%)	92 (76.7%)	>0.999
Age (SD)	26.42 (4.54)	27.18 (4.56)	0.294
Years of Education (SD)	15.80 (1.36)	16.02 (1.68)	0.388
Physical characteristics			
BMI (SD)	24.26 (5.07)	24.73 (7.98)	0.865
BMI < 25	37 (61.6%)	73 (60.8%)	0.100
Overweight	13 (21.7%)	38 (31.7%)	
Obese	10 (16.7%)	9 (7.5%)	
Body fat percentage (SD)	23.35 (7.78)	23.80 (5.77)	0.610
SBP, mmHg (SD)	114.15 (12.23)	112.47 (18.99)	0.476
DBP, mmHg (SD)	76.03 (10.23)	73.69 (10.23)	0.134
HDL, mg/dL (SD)	50.41 (11.58)	52.29 (13.27)	0.352
LDL, mg/dL (SD)	111.62 (32.02)	113.35 (35.45)	0.750
Total cholesterol, mg/dL (SD)	181.83 (30.77)	187.28 (35.61)	0.317
TG, mg/dL (SD)	143.29 (165.68)	116.24 (77.28)	0.313
Psychiatric characteristics			
CIAS (SD)	79.48 (11.62)	40.22 (14.70)	**<0.001**
CSM (SD)	25.22 (6.56)	34.91 (6.67)	**<0.001**
DSPS (%)	32 (53.33%)	11 (9.17%)	**<0.001**
Chronotype (%)			**<0.001**
Morningness	0 (0%)	14 (11.7%)	
Intermediate	38 (63.3%)	101 (84.2%)	
Eveningness	22 (36.7%)	5 (4.2%)	
PIRS (SD)	28.85 (10.88)	14.88 (9.48)	**<0.001**
Insomnia (%)	17 (28.33%)	8 (7.14%)	**<0.001**

GD, gaming disorder; SD, standard deviation; BMI, body mass index; SBP, systolic blood pressure; DBP, diastolic blood pressure; HDL, high-density lipoprotein; LDL, low-density lipoprotein; TG, triglycerides; CIAS, Chinese Internet Addiction Scale; DSPS, delayed sleep phase syndrome; CSM, composite scale of morningness; PIRS, Pittsburg insomnia rating scale. Bold type indicates *p* < 0.05.

**Table 2:** Physical activity markers of participants with and without gaming disorder.

	GD Group (*n* = 34)	Control Group (*n* = 39)	*p* Value
Daily calorie expenditure, kcal (SD)	305.94 (150.54)	469.69 (159.02)	**<0.001**
Daily steps (SD)	5865.74 (2892.53)	8865.00 (2904.64)	**<0.001**
Daily walking distance, km (SD)	4.64 (2.30)	7.15 (2.45)	**<0.001**
Time to fall sleep, min (SD)	134.09 (70.74)	65.15 (60.11)	**<0.001**
Total sleep, min (SD)	446.18 (71.89)	417.95 (51.84)	0.057

GD, gaming disorder; SD, standard deviation; kcal, kilocalorie; km, kilometer; min, minutes. Bold type indicates *p* < 0.05.

**Table 3:** Demographic, physical, and psychiatric characteristics of participants with gaming disorder with and without delayed sleep phase syndrome.

	DSPS Group (*n* = 32)	Non-DSPS Group (*n* = 28)	*p* Value
Demographic characteristics			
Male (%)	23 (71.9%)	23 (82.1%)	0.348
Age (SD)	25.31 (4.02)	27.68 (4.83)	**0.043**
Years of education (SD)	15.81 (1.09)	15.79 (1.64)	0.940
Physical characteristics			
BMI (SD)	22.33 (4.04)	26.47 (5.29)	**0.001**
BMI < 25	25 (78.1%)	12 (42.9%)	**0.013**
Overweight	5 (15.6%)	8 (28.6%)	
Obese	2 (6.3%)	8 (28.6%)	
Body fat percentage (SD)	22.03 (7.35)	24.85 (8.11)	0.162
SBP, mmHg (SD)	110.36 (10.28)	118.46 (13.01)	0.090
DBP, mmHg (SD)	74.22 (8.25)	78.11 (9.43)	0.094
HDL, mg/dL (SD)	51.18 (11.67)	49.53 (11.63)	0.584
LDL, mg/dL (SD)	104.84 (24.70)	119.36 (37.73)	0.079
Total cholesterol, mg/dL (SD)	174.78 (28.32)	189.89 (31.97)	0.057
TG, mg/dL (SD)	148.66 (214.68)	137.14 (83.62)	0.791
Psychiatric characteristics			
CIAS (SD)	80.32 (12.59)	78.75 (10.85)	0.605
CSM (SD)	22.06 (4.75)	28.82 (6.54)	**<0.001**
Chronotype			**0.005**
Intermediate (%)	15 (46.9%)	23 (82.1%)	
Eveningness (%)	17 (53.1%)	5 (17.9%)	
PIRS (SD)	30.09 (10.47)	27.43 (11.34)	0.348
Insomnia (%)	14 (43.8%)	3(10.7.%)	**0.005**

SD, standard deviation; DSPS, delayed sleep phase syndrome; BMI, body mass index; SBP, systolic blood pressure; DBP, diastolic blood pressure; HDL, high-density lipoprotein; LDL, low-density lipoprotein; TG, triglycerides; CIAS, Chinese Internet Addiction Scale; CSM, composite scale of morningness; PIRS, Pittsburg insomnia rating scale. Bold type indicates *p* < 0.05.

**Table 4:** Physical activity markers of participants with gaming disorder with and without delayed sleep phase syndrome.

	DSPS Group (*n* = 18)	Non-DSPS Group (*n* = 16)	*p* Value
Daily calorie expenditure, kcal (SD)	316.8 (153.93)	293.68 (150.65)	0.661
Daily steps (SD)	6074.90 (2962.98)	5630.44 (2888.84)	0.662
Daily walking distance, km (SD)	4.80 (2.35)	4.46 (2.30)	0.665
Time to fall sleep, min (SD)	140.76 (52.02)	127.41 (86.72)	0.591
Total sleep, min (SD)	462.59 (72.74)	429.65 (69.23)	0.186

DSPS, delayed sleep phase syndrome; SD, standard deviation; kcal, kilocalorie; km, kilometer; min, minutes.

## Data Availability

The data is unavailable due to privacy and ethical restrictions.
